# Role of polysaccharide structure in the rheological, physical and sensory properties of low-fat ice cream

**DOI:** 10.1016/j.crfs.2023.100531

**Published:** 2023-06-11

**Authors:** Xiangyu Liu, Guido Sala, Elke Scholten

**Affiliations:** Physics and Physical Chemistry of Foods, Wageningen University, Bornse Weilanden 9, 6708 WG, Wageningen, the Netherlands

**Keywords:** Polysaccharide flexibility, Serum phase viscosity, Ice cream structure, Sensory perception

## Abstract

Polysaccharides can be used as fat replacers in ice cream, as they contribute to an increase of viscosity. However, no research has clarified the exact role of viscosity from that of the structure of the polysaccharides on the properties of ice cream. In this study, the effect of polysaccharide structure on different properties of low-fat ice cream was investigated. The polysaccharides taken into consideration varied from flexible (locust bean gum and guar gum) to rigid (xanthan gum and iota carrageenan). Relationships between rheological properties of ice cream mixes and microstructural characteristics and sensory perception of the final ice cream were established. To separate the effect of the polysaccharide structure from that of viscosity, two series of ice cream were prepared: one in which the mix viscosity of the various samples was similar (approximately 68.3 mPa· s), and one in which the serum phase viscosity was similar (approximately 15563 mPa· s). Flexible polysaccharides showed a lower degree of shear-thinning and a more liquid-like viscoelastic behavior compared with rigid polysaccharides. In addition, flexible polysaccharides led to higher overrun (47–58%) than other samples (approximately 30%), which resulted in lower hardness of the ice cream (<3.2 MPa). Rigid polysaccharides caused gelation of the serum phase, which made the ice cream more difficult to scoop. Based on the results of the sensory evaluation, flexible polysaccharides could provide higher softness and creaminess-related properties, while rigid polysaccharides resulted in higher coldness and grittiness. Therefore, polysaccharides with a flexible structure are a better choice for improving the textural and sensory properties of low-fat ice cream.

## Highlights

• Effect of polysaccharide on ice cream properties was separated from that of viscosity.
• Flexible polysaccharides led to higher overrun and lower hardness of ice cream.
• Melting properties were found to relate more to serum phase viscosity.
• Flexible polysaccharides could provide higher softness and creaminess.
• Rigid polysaccharides resulted in higher coldness and grittiness.

## Graphical abstract

**Figure:**
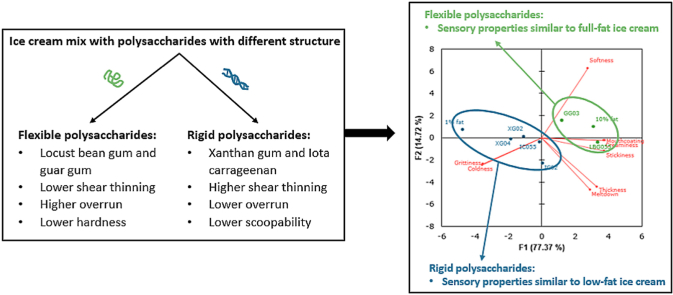

## 1 Introduction

Ice cream is a popular dessert and is highly liked by consumers. However, it contains high amounts of fat, and an excessive consumption of high-fat foods is linked nowadays to obesity and cardiovascular disease, diabetes and cancer. The microstructure of ice cream is rather complex, consisting of air cells, ice crystals, and a network of coalesced fat droplets entrapped in a thick continuous phase (Scholten, 2014). Commercial ice cream contains 10–16% dairy fat, which is an important component in affecting melting rate, shape retention after melting, overrun, creaminess and mouthcoating, which can be attributed to the formation of a fat network in the unfrozen serum phase and the lubricating effect of fat (Liu et al., 2022; Milani and Koocheki, 2011; Roland et al., 1999; Rolon et al., 2017). Although low-fat ice creams are already available on the market, such products still have lower quality. Therefore, fat reduction in ice cream while retaining the sensory quality of the full-fat product still remains a challenge, and requires ingredients able to replace the functionalities of fat.

Based on their composition, fat replacers are mainly categorized into three groups: lipid-, protein- and carbohydrate-based (Akbari et al., 2019). Carbohydrate-based fat replacers are most common, and many polysaccharides have already been used also in ice cream. These polysaccharides contribute largely to viscosity enhancement and give ice cream a creamy and full mouthfeel (Akbari et al., 2019). Many studies have shown that the addition of polysaccharides in low-fat ice cream can be an effective way to improve the physical and sensory properties of low-fat or fat-free ice cream (Javidi et al., 2016; Kurt et al., 2016; Mahdian and Karazhian, 2013; Soukoulis et al., 2008). Up to now, mainly polysaccharides have been subject of investigation for their effect on the rheological properties of ice cream mixes, and mix viscosity is considered to be the main factor in affecting the ice cream microstructure and sensory properties. For example, an increase in viscosity has been shown to have a positive effect on overrun, melting resistance and sensory properties (BahramParvar et al., 2013; Mahdian et al., 2013; Milani et al., 2011). In addition, shear thinning and thixotropy of ice cream mixes have been related to an enhancement of creaminess and a reduction of coldness and coarseness (Javidi et al., 2016). However, in these studies the viscosity of the ice cream mixes with similar concentration was not constant, as the structure of the polysaccharides has an influence on the rheological profile. This makes it difficult to distinguish the exact role of viscosity from that of the structure of the polysaccharides in the different structural and physical properties of ice cream. For a better understanding of these aspects, mix viscosity should be kept constant by varying the concentration of different polysaccharides. In addition, the structure of polysaccharides may also affect serum phase viscosity, which refers to the viscosity of the unfrozen continuous phase excluding air cells and ice crystals. The serum phase viscosity originates from the mix viscosity and undergoes a significant increase during the freezing process, as the concentration of different soluble components within the unfrozen phase becomes more and more pronounced due to ice crystallization. However, for sensory perception, the viscosity of the serum phase resulting from freeze concentration may become more relevant than mix viscosity, as the mouthfeel of ice cream is more related to its frozen form (Goff and Hartel, 2013). To clarify the exact role of both mix viscosity and serum phase viscosity on the different properties of ice cream, samples with the same serum phase viscosity should also be compared.

Besides viscosity, the sensory perception of ice cream may also be related to the lubrication properties of the different polysaccharides. Even though studies on the tribological properties of individual polysaccharides have been carried out (Ji et al., 2022; Stokes et al., 2011; Taira and McNamee, 2014), the lubrication functionality of these molecules in ice cream is still unclear. It has been reported that rigid polysaccharides like xanthan provide better lubrication than flexible polymers like guar gum, although the viscosity of the samples was the same (Ji et al., 2022). It is not clear how these differences may affect the perception of ice cream, and which sensory attributes may be influenced by lubrication. A better understanding of the exact role of the structure of the polysaccharides in structural, tribological, rheological and sensory properties of ice cream is still required.

Our study aimed to shed light on the described issues by investigating the addition of a set of polysaccharides varying in molecular flexibility in fat-reduced ice cream. Two reference samples with 1% and 10% fat were used. Four polysaccharides differing in structure were used to gain insight in how both mix and serum phase viscosity relates to different structural and sensory aspects: locust bean gum, guar gum, xanthan gum and iota-carrageenan. Both locust bean gum (LBG) and guar gum (GG) have a random coil conformation, with a persistence length of approximately 3 nm, and are therefore considered flexible polysaccharides (Picout et al., 2002). Xanthan gum (XG) has a rigid-rod conformation, with a persistence length of 5–35 nm, and is therefore more rigid than LBG and GG. Iota-Carrageenan (IC) has a rigid conformation, with a persistence length of 23–26 nm (Thrimawithana et al., 2010). Our samples set thus included two random coil polysaccharides (LBG and GG) and two rigid polysaccharides (XG and IC). The differences in morphology of these four polysaccharides have been widely discussed in literature (Dogan et al., 2018; Ferdiansyah et al., 2023; Hadinugroho et al., 2021). To be able to isolate the effect of polysaccharide structure, we prepared two series of ice cream, one in which the mix viscosity was matched, and one in which the serum phase viscosity of the ice cream was matched. We determined the rheological behavior of these different polysaccharides (consistency coefficient, flow behavior index, and loss factor) in ice cream mixes, which was then linked to the structure of the obtained ice creams (overrun, air cell size, ice crystal size, serum phase viscosity). The ice cream structure was subsequently linked to different rheological (G' and G" upon temperature increase), textural (hardness and scoopability), lubrication, and sensory properties (such as hardness, coldness and creaminess) of the samples.

## 2 Materials and methods

### 2.1 Materials

Organic carrageenan-free cream (33% fat, 3% lactose, 2.4% protein, 0.08% minerals), organic skimmed milk (5% lactose, 3.5% protein, 0.1% minerals) and sucrose were purchased from a local supermarket (Jumbo Wageningen, Netherlands). Locust bean gum (LBG,∼ 310 kDa), guar gum (GG,∼ 4000 kDa), xanthan gum (XG,∼ 4000 kDa), iota carrageenan (IC,∼ 120 kDa) were purchased from Sigma-Aldrich Chemie GmbH (Steinheim, Germany). Vanillin (100%) was purchased from Royal Polak Spices (Steenwijk, Netherlands).

### 2.2 Ice cream mix preparation

Two different series of low-fat ice cream (1% fat) were prepared by using different polysaccharides. In one series, we matched the mix viscosity measured at 50 s^-1^ by varying the concentration of different polysaccharides (0.55% LBG, 0.3% GG, 0.2% XG and 0.2% IC). In the other series, the serum phase viscosity measured at the mentioned shear rate and at -20 °C was matched in the same way (0.55% LBG, 0.3% GG, 0.4% XG and 0.55% IC). The sugar concentration in the continuous phase of both series was kept the same (∼15%) to guarantee constant freezing point depression and ice fraction. Vanillin (0.1%) was added to all samples. In addition, two reference samples were prepared, both without polysaccharides: a low-fat ice cream with 1% fat, and a high-fat ice cream with 10% fat. An overview of the model ice cream recipes is shown in Table 1.

**Table 1:** Ice cream formulations of the studied samples (LBG: locust bean gum; GG: guar gum, XG: xanthan gum; IC: iota carrageenan).

Ingredients (%)	10% fat	1% fat	LBG	GG	XG		IC	
Cream	30	3.0	2.98	2.99	2.99	2.99	2.99	2.98
Skimmed milk	56.2	81.9	81.47	81.67	81.75	81.59	81.75	81.47
Sucrose	13.8	15.0	14.90	14.94	14.96	14.93	14.96	14.90
Vanillin	0.1	0.1	0.1	0.1	0.1	0.1	0.1	0.1
Polysaccharide (similar mix viscosity)	0	0	0.55	0.3	0.2	0	0.2	0
Polysaccharide (similar serum phase viscosity)	0	0	0.55	0.3	0	0.4	0	0.55

For the preparation of the ice cream mixes, sugar and skimmed milk were first mixed with a magnetic stirrer for 30 min. Then, different amounts of polysaccharides and vanillin were added slowly upon stirring at approximately 25 °C for 1.5 h. The mixed systems were then heated in a water bath at 85 °C for 30 min, to achieve complete dissolution of the polysaccharides. The mixes were cooled to room temperature (approximately 25 °C) and cream was added upon stirring for 1 h before ice cream preparation. The mixes were aged at 4 °C overnight.

### 2.3 Ice cream preparation

Ice cream samples (3 L) were frozen in a batch freezer (Frigomat T4S-T5S, Italy) for 8 min and the draw temperature was approximately -5 °C. The ice cream was collected into different containers depending on the determination to be carried out: plastic rings of 25 mm in height and 70 mm in diameter for hardness and scoopability measurements, metal rings of 5 mm in height and 25 mm in diameter for rheological characterization, and 60 mL containers for sensory evaluation. These samples were first hardened at -20 °C for 24 h to solidify further before measurement.

### 2.4 Determination of the rheological properties of ice cream mixes

#### 2.4.1 Ice cream mix viscosity

The viscosity of ice cream mixes was measured using a rheometer (MCR 501, Anton Paar, Germany) equipped with a concentric cylinder geometry (probe CC17/Ti; cup CC17/Ti). A sample of 4.7 ml was added to the geometry and the temperature was set at 20 °C. The viscosity was measured at a shear rate ranging from 0.1 to 300 s^-1^ in a time frame of 5 min. The flow behavior of the ice cream mix was described by a power law model as:

(1) $$\sigma =K\gamma^{n}$$

where σ is the shear stress (Pa), K is the consistency coefficient (Pa·s^n^, equal to viscosity when n = 1), γ is the shear rate (s^-1^) and n, the flow behavior index, is a dimensionless number reflecting the shear-thinning behavior. Lower values of n represent a higher degree of shear-thinning behavior. The measurements were repeated three times for each sample.

#### 2.4.2 Viscoelastic properties of ice cream mix

The viscoelastic properties (G' and G") of the ice cream mixes were measured with a rheometer (MCR 501, Anton Paar, Germany). Prior to measurements, the samples (2 ml) were placed into a PP50 plate/plate geometry and allowed to equilibrate at 20 °C for 120 s. The strain was set at 0.5% and the frequency at 1.6 Hz. Storage modulus (G') and loss modulus (G") were measured during a time frame of 5 min. The measurements were repeated three times for each sample. The mean value of G' of the ice cream mix and the mean value of loss factor of mix (G’’/G') were extracted to characterize the viscoelastic behavior of ice cream mixes.

### 2.5 Determination of structural elements of ice cream

#### 2.5.1 Overrun of frozen ice cream

The overrun of frozen ice cream was determined by first weighing a fixed volume (30 ml) of the aged pre-mix in a metal cup. Next, the same volume of ice cream was weighted in the cup directly after preparation. The overrun was quantified as Muse and Hartel (2004):

(2) $$Overrun\;\left(\%\right)=\frac{Weight\;of\;mix-weight\;of\;ice\;cream}{Weight\;of\;ice\;cream}\times 100$$

#### 2.5.2 Ice crystal and air cell size of frozen ice cream

The analysis of ice crystal size and air cell size was performed according to the methods described by Velásquez-Cock et al. (2019) and Muse et al. (2004), respectively, with slight modifications. A light microscope with a hot stage (Zeiss Axioskop 2 Plus, Germany) was used, which allowed to control the temperature of the microscope at -20 °C. For the analysis of ice crystal size, all tools, reagents and samples were kept at -20 °C before sample preparation. Five mg of ice cream sample were taken from the core section of each container with a sharp knife and deposited over a standard glass slide. One or two drops of kerosene (approximately -20 °C) were applied to disperse the ice crystals more evenly, and the glass slide was covered with a chilled cover slide. Ice crystals were spread out gently by tapping the cover slide with chilled tweezers. The whole sample preparation process was carried out in a -20 °C storage room to prevent melting of the ice crystals.

For the analysis of air cell size, a small sample of ice cream was placed on a prechilled microscope slide within an area delimited by a plastic frame (65 μl, 1.5 x 1.6 cm) to create a space to prevent the deformation of air cells. The slides were sealed with a cover slide to smear the sample into a thin layer and prevent the escape of air cells. The temperature of the hot stage was adjusted to -6 °C, and at this temperature the air cells were sufficiently buoyant to rise to the bottom of the top slide for size determination.

Images of both ice crystals and air cells were taken at 10x magnification to include at least 300 ice crystals or air cells to calculate mean size and standard deviation. The size of ice crystals and air cells was determined by placing a circle around an ice crystal or air cell manually, from which the area and radius were calculated using the software ZEN 2011 assuming that the crystals and air cells were of spherical shape.

#### 2.5.3 Serum phase viscosity of frozen ice cream

The serum phase viscosity in ice cream cannot be measured directly in the frozen state. Therefore, the serum phase of the various samples was mimicked taking into account the amount of polysaccharides, sugar and other ingredients in the non-frozen fraction. First, the amount of unfrozen water was calculated based on the measured ice fraction. The ice fraction as determined by the Differential Scanning Calorimetry (DSC) was approximately 62% in all samples. Based on this, the concentrations of water-soluble ingredients (polysaccharides, sucrose, lactose, protein and salt) in the unfrozen serum phase were calculated. These concentrations were used to recreate the unfrozen serum phase. The exact recipes of the recreated serum phase of all samples can be seen in Table 2.

**Table 2:** Unfrozen serum phase formulations of the studied samples. (LBG: locust bean gum; GG: guar gum, XG: xanthan gum; IC: iota carrageenan).

Ingredients (%)	10% fat	1% fat	LBG	GG	XG		IC	
Sucrose	38.59	38.65	38.12	38.35	38.45	38.26	38.45	38.12
SMP	21.76	21.64	21.34	21.47	21.53	21.42	21.53	21.34
Water	39.65	39.71	39.16	39.41	39.51	39.31	39.51	39.16
Polysaccharide (similar mix viscosity)	0	0	1.40	0.77	0.51	0	0.51	0
Polysaccharide (similar serum phase viscosity)	0	0	1.40	0.77	0	1.02	0	1.40

The serum phase viscosity measurements were performed using a rheometer MCR 501 equipped with a CC17/Ti geometry. During the measuring process, the temperature was decreased from 4 to -20 °C with a cooling rate of 0.5 °C/min. The shear rate was kept constant at 50 s^-1^. In some cases, ice crystal formation and growth were still observed, and therefore not all samples could be measured over the entire temperature range. In these cases, the serum phase viscosity was estimated by extrapolation of the results to -20 °C.

#### 2.5.4 Evaluation of network formation in the serum phase

To evaluate the formation of a fat network, the particle size distribution of ice cream mix and molten full-fat ice cream was measured with static light scattering (Mastersizer 3000, Malvern Instrument, Ltd, Malvern, Worcestershire, UK), using a refractive index of 1.46 and 1.33 for the fat and the water, respectively. A bimodal distribution was observed in the molten ice cream, and according to our previous work (Liu et al., 2022), the second peak with a particle size higher than 10 μm could be regarded as aggregated fat clusters. The mean particle size of ice cream mix and molten ice cream (D_4,3_) was determined separately, and the volume percentage of the aggregated fat clusters was used to reflect the degree of fat destabilization as fat aggregate percentage. To evaluate possible changes in microstructure due to the formation of a polysaccharide, the viscosity of the molten ice cream was compared to viscosity measured before ice cream preparation. The viscosity of the molten ice cream samples was measured at a shear rate between 0.1 and 100 s^-1^ with a rheometer (Physica MCR 501, Anton Paar, Germany) equipped with the cup geometry (CC17/T200/Ti). The temperature was set at 20 °C and the total measuring time was set at 5 min. The measurements were repeated three times for each sample. Based on our measurements, both the viscosity of the ice cream mix and that of the molten ice cream showed shear-thinning behavior, indicating that the structure of the samples changed during the shearing process. We took the viscosity of both the mix and molten ice cream at low shear rate (0.1 s^-1^) to represent the structure of the samples, and their ratio was used to characterize network formation by fat aggregates or polysaccharides, with low values indicating a higher degree of network formation.

### 2.6 Physical and rheological properties

#### 2.6.1 Hardness and scoopability of frozen ice cream

Hardness and scoopability of the samples were measured with a Texture Analyzer (TA-TX plus, Stable Micro Systems, UK). Samples with fixed volume were prepared using plastic rings of 25 mm in height and 70 mm in diameter. The samples were stored in a freezer at -20 °C for a minimum of 24 h. Prior to measurement, a climate chamber was connected to the Texture Analyzer to maintain a temperature of -20 °C. For hardness determination, the samples were taken out of the plastic rings and were transferred to the climate chamber immediately (-20 °C). Then they were penetrated with an aluminum cylinder probe (5 mm in diameter) attached to a 50 kg load cell to a strain of 50% at a speed of 2 mm/s. Hardness was taken as the maximum stress of the stress-strain curve.

**Fig. 1:**
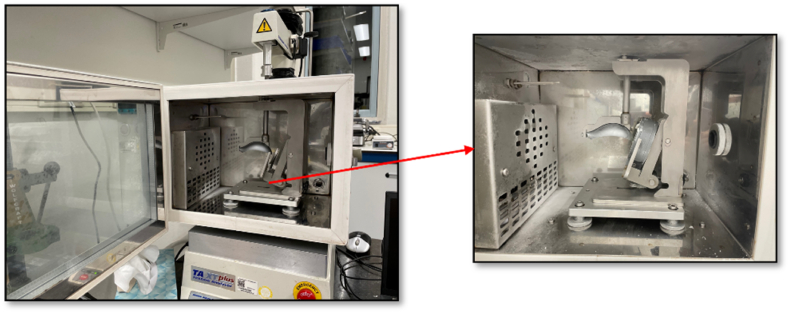
Climate chamber with the scoopability measurement setup. The dimensions of the ring: 25 mm in height and 70 mm in diameter; dimensions of the spoon: 63 x 33 x 25 mm. Angle of the ring: 65°.

To measure scoopability, a scooping spoon probe in combination with an ice cream sample holder was used (see Fig. 1), which were mounted in a climate chamber hold at a temperature of -20 °C with liquid nitrogen. The scooping spoon was forced into the ice cream with a constant velocity of 3 mm/s over a distance of 25 mm. We used the total area under the curve to determine the scooping energy (N · mm), which we used as a measure for scoopability: the larger the scooping energy, the lower the scoopability. Both hardness and scoopability measurements were done in triplicate to obtain average value and standard deviation.

#### 2.6.2 Melting properties of frozen ice cream

Temperature sweeps were performed to explore the melting process of the studied ice cream samples by increasing the temperature from -20 to 10 °C. Measurements were performed using a rheometer (MCR 301, Anton Paar, Germany) at a constant strain of 0.005% and a frequency of 1.6 Hz with a plate-plate geometry (PP50/P2). A moveable hood covering the plate-plate geometry was connected to the cooling system to control the temperature. An air pump was also connected to the hood to prevent heat exchange with the environment. Prior to the measurement, the initial temperature of the plate was reduced to -20 °C using a Peltier element, and then the temperature was increased to 10 °C with a heating rate of 0.5 °C/min. Sixty measuring points were recorded and the total measuring time was 60 min. Storage modulus (G') and loss modulus (G") were measured. Three zones could be identified during the whole process (Wildmoser et al., 2004) and two parameters were extracted after the measurements: (1) the mean value of G' in the frozen state (zone I, G'ZI), and (2) the slope of G' during the melting stage (zone II, SZII), which is an indicator for the speed of melting (Eisner et al., 2005). Ice cream samples were measured at least twice to obtain average values.

#### 2.6.3 Lubrication behavior of molten ice cream

Tribology measurements were carried out to evaluate the lubrication properties of molten ice cream samples. Measurements were performed with a MCR 301 rheometer equipped with a tribology accessory (T-PTD 200, BC 12.7, Anton Paar, Austria). The set-up was based on a glass ball-on-three-pins principle, consisting of a spherical glass ball (d = 12.7 mm) and three PDMS pins (d = 6 mm, roughness 0.2 μm ± 0.03). The temperature of the tribology cup was set at 20 °C and a normal force of 1 N was applied. Samples with a volume of 0.6 ml were poured into the tribology cup, and the friction coefficient was measured as a function of sliding speed. The measurements consisted of two runs in total with increasing and decreasing sliding speed between 0.01 and 470 mm/s, in 7.5 min per run. The data of the second run with increasing speed showed good repeatability and were selected for further analysis. The measured friction coefficient was plotted versus sliding speed. Two regimes could be identified: a boundary regime at low sliding speed, and a mixed regime at intermediate sliding speeds. A hydrodynamic regime was not obtained as the viscosities of the samples were not high enough. The mean friction coefficient in the boundary regime (FCB) and the slope of friction coefficient over sliding speed in the mixed regime (SMR) were used to represent different lubrication properties of molten ice cream samples.

### 2.7 Sensory analysis

A sensory study was performed using an untrained panel (n = 80 participants; gender: 43 female, 37 male; age range: 18 - 32). Participants were recruited from the campus of Wageningen University & Research. They all had good general and oral health, good/normal tasting and smelling abilities, used no medication and had no allergies or intolerances towards the ingredients used in the samples. A consent form was signed by the participants and they were given a financial compensation upon completion of the study.

A rate-all-that-apply (RATA) methodology was applied, based on previous works in which structural aspects were related to sensory perception for foods in which differences were limited (Fuhrmann et al., 2020; Ji, den Otter, Cornacchia, Sala and Scholten, 2023; Meyners et al., 2016). The samples were stored in the freezer (-20 °C) and coded with 3-digit numbers according to a random design. Each sample was supplied in individual plastic containers of 60 ml and kept at room temperature (approximately 25 °C) for 5 min before serving. For logistics reasons, the participants were divided into 8 groups, and the serving order of samples was randomized for each group using a Latin Square design to prevent presentation order effects. The provided attribute list and definition are shown in Table 3. Attributes and definitions were taken from previous works (Alvarez, 2009; Pintor et al., 2017; Thompson et al., 2009) and adjusted when necessary. The participants selected the attributes that were applicable to their samples (creaminess, softness, coldness, grittiness, thickness, stickiness, mouth coating, meltdown, off-flavor) and when selected, these attributes were scored on a 1-to-9 scale, with anchors “weak” to “strong”. In addition, participants were also asked to rate their overall liking of each sample using a 9-point hedonic scale (“Dislike extremely” to “Like extremely”). Between samples, the participants were instructed to drink water to remove the residue of the previous sample from the mouth.

**Table 3:** Sensory attributes and definitions for the sensory test.

Attribute	Definition
Creaminess	Refers to the intensity of the “fatty” feeling in the mouth when the sample is manipulated between the tongue and the palate; perceived fat content
Softness	The ease of compressing the sample between the tongue and palate
Coldness	The feeling of cold in the mouth/upper gastrointestinal tract upon eating or swallowing the sample
Grittiness	The immediate perception of crystal-like particles within the sample
Thickness	The pressure necessary to move the sample between the tongue and the palate
Stickiness	The elasticity between the tongue and the palate when coated with the sample
Mouth coating	A sensation of having a coating on the tongue and other mouth surfaces
Meltdown	The time required for the product to melt in the mouth when continuously pressed by the tongue against the palate
Off-flavor	An unpleasant or unusual flavor that is not typically associated with ice cream
Overall liking	How much was the sample liked

### 2.8 Statistical analysis

The data obtained from rheological, tribological and textural measurements were analyzed with SPSS software (Version 25.0, IBM Corp). The means were compared with a Tukey’s test at a 5% level of significance using an analysis of variance (ANOVA). Moreover, RATA data and overall liking were analyzed using linear mixed models using RStudio with the additional packages LmerTest and Emmeans (RStudio, Inc., Version 4.0.2). Significance level of P < 0.05 was chosen. Additionally, the results obtained from rheological, physical and sensory measurements were analyzed by Principal Component Analysis (PCA) and correlation matrices were established for the different properties. In addition, biplots obtained from PCA were used to visually represent sample grouping and differentiation. PCA analyses were performed using XLSTAT software (Addinsoft, Paris, France).

## 3 Results and discussion

### 3.1 Effect of polysaccharide structure on rheological properties of ice cream mix and serum phase

We first investigated the effect of polysaccharides on the rheological properties of ice cream mixes. As shown in Table 4, both the high-fat sample and the low-fat sample, without polysaccharides, had a lower viscosity of 6.8 and 3.3 mPa· s, respectively. Upon addition of polysaccharides, the viscosity increased, and the extent of the increase depended on the type of polysaccharide. For the series with mix viscosity matched at 50 s^-1^, 0.55% of LBG and 0.3% of GG were used to obtain a viscosity of approximately 70 mPa· s, whereas only 0.2% of XG and IC were needed to achieve the same viscosity value. This can be explained by the molecular weight and structure of the various polysaccharides. Both LBG and GG have a flexible structure, but the GG used for our research had a molecular weight higher (∼ 4000 kDa) than that of LBG (∼ 310 kDa). Therefore, a lower concentration of GG was required to achieve the desired viscosity. However, the polysaccharide structure seemed to be more important than molecular weight in affecting viscosity. As a matter of fact, a comparable low concentration for XG and IC was required to obtain the desired viscosity (70 mPa· s), even though their molecular weight was quite different (XG: 4000 kDa; IC: 120 kDa). Both XG and IC are more rigid than GG and LBG; they were more effective in increasing viscosity, and therefore their concentration was lower. However, even though the viscosity values were matched, consistency coefficient (K) and flow behavior index (n) were very different, indicating a different shear-thinning behavior for the different polysaccharides. A higher K could be observed in the XG and IC samples, which also proved the higher ability of these polysaccharides to increase viscosity. In addition, smaller n values were found in the samples containing rigid polysaccharides, indicating a greater degree of shear thinning (Rosti and Takagi, 2021). This can be explained by the fact that rigid polysaccharides can be more easily aligned in the direction of the shear flow (Bai et al., 2017). The difference in structure also had an influence on serum phase viscosity. Even though the mix viscosity was similar, the serum phase viscosity of ice creams with rigid polysaccharides was lower. This may be attributed to changes in solvent quality. Upon freezing, the concentration of polysaccharides and all solutes (such as ions and milk proteins) in the serum phase increased. This may lower the solvent quality for both XG and IC, reducing their ability to contribute to viscosity. In addition, screening of the charged groups by ions might reduce the rigidity of anionic polysaccharides, although this is not commonly observed for rigid molecules. Such a change in solubility or rigidity would explain the decrease in serum phase viscosity. This result is in contradiction with the statement of Amador et al. (2017), who reported that serum phase viscosity was expected to be higher in samples with rigid polysaccharides. However, in their study, they did not measure the serum phase viscosity, but based their expectation on extrapolations. Our results show that such extrapolation cannot be used, and that the structure of the polysaccharides plays a large role in these phenomena.

**Table 4:** Rheological properties of ice cream mix samples with different polysaccharides. The notation of the samples includes the name of the polysaccharide and the concentration used. For example, LBG055 refers to a sample with 0.55% LBG.

Ice cream mix	Mix viscosity (mPa·s)	Serum phase viscosity (mPa·s)	Mix consistency coefficient (K)	Mix flow behavior index (n)	Mix G' (Pa)	Mix loss factor (G"/G')
1% fat	3.3 ± 0.4^d^	2579 ± 655^c^	3.8 ± 0.3^f^	0.98 ± 0.02^a^	0.9 ± 0.3^d^	0.83 ± 0.02^b^
10% fat	6.8 ± 0.1^d^	2608 ± 496^c^	10.8 ± 1.1^f^	0.92 ± 0.02^a^	7.5 ± 0.5^bcd^	0.61 ± 0.07^c^
LBG055^a^^,^^b^	68.3 ± 1.9^c^	15563 ± 2338^a^	137.2 ± 8.2^e^	0.80 ± 0.01^b^	4.2 ± 0.7^bcd^	0.97 ± 0.01^a^
GG03^a^^,^^b^	70.6 ± 1.4^c^	16741 ± 1478^a^	237.9 ± 7.3^d^	0.67 ± 0.03^c^	2.7 ± 0.5^cd^	0.91 ± 0.02^ab^
XG02^a^	66.6 ± 1.8^c^	7666 ± 1306^b^	424.9 ± 10.2^c^	0.51 ± 0.04^d^	9.0 ± 1.1^bc^	0.48 ± 0.02^d^
IC02^a^	68.1 ± 2.1^c^	4136 ± 641^bc^	760.2 ± 9.8^b^	0.42 ± 0.02^d^	12.1 ± 0.5^b^	0.28 ± 0.04^e^
XG04^b^	398.5 ± 2.5^a^	14669 ± 1517^a^	3221.5 ± 14.5^a^	0.41 ± 0.02^d^	20.3 ± 3.1^a^	0.36 ± 0.04^de^
IC055^b^	211.2 ± 3.1^b^	15388 ± 2145^a^	2544.1 ± 13.9^a^	0.37 ± 0.01^d^	21.5 ± 0.5^a^	0.25 ± 0.04^e^

Values with a different letter within the same column are significantly different (P < 0.05).

a Group of ice cream mixes with similar mix viscosity.

b Group of ice cream mixes with similar serum phase viscosity.

To obtain the series with matched serum viscosity, we adjusted the concentrations of the polysaccharides. We took the sample containing 0.55% LBG as a reference. Since the molecule flexibility of GG is similar to that of LBG, the serum phase viscosity of samples containing these ingredients was also similar, and the concentration of GG did not have to be adjusted. However, in the case of XG and IC, to achieve a comparable serum phase viscosity as LBG and GG, their concentration had to be increased to 0.4% and 0.55%, respectively. Based on our findings, we can conclude that the serum phase viscosity and the link between mix viscosity and serum phase viscosity mainly depend on the structure of the polysaccharides.

The storage modulus (G') and loss factor (G"/G') of ice cream mixes were also measured to characterize the viscoelastic properties of ice cream mixes. As shown in Table 4, ice cream mixes with flexible polysaccharides (LBG055 and GG03) showed a relatively lower G' and a significantly higher loss factor than mixes with rigid polysaccharides (XG and IC), indicating that more flexible polysaccharides gave a more liquid-like behavior. In contrast, XG and especially IC showed higher G', i.e., more solid-like properties, which were most likely related to the ability of these stiff polysaccharides to form a gel at the chosen concentrations (Petri, 2015; Thrimawithana et al., 2010). This could be partly explained by their negative charge. As both cream and skim milk contain divalent calcium ions, a XG or IC network may have formed through salt bridges.

### 3.2 Properties of ice cream

#### 3.2.1 Microstructural characteristics

As shear-thinning behavior and viscoelastic properties of the ice cream mixes were expected to directly affect the structure of the frozen ice cream (air phase, ice phase and serum phase), overrun, air cell size, ice crystal size and serum phase viscosity were analyzed, and the results are summarized in Table 5. To quantify a possible network formation during the freezing process due to fat aggregation or polysaccharide gelation, we also compared the viscosity of the final molten ice cream with that of the initial ice cream mix at a low shear rate.

**Table 5:** Microstructural characteristics of ice cream samples with different polysaccharides. The notation of the samples includes the name of the polysaccharide and the concentration used. For example, LBG055 refers to a sample with 0.55% LBG.

Ice cream	Overrun (%)	Air cell size (μm)	Ice crystal size (μm)	Mix viscosity/viscosity of molten ice cream
1% fat	34 ± 4^cd^	50 ± 27^a^	55 ± 18^a^	0.87 ± 0.08^a^
10% fat	32 ± 1^d^	60 ± 27^a^	57 ± 17^a^	<0.01
LBG055^a^^,^^b^	47 ± 2^b^	52 ± 22^a^	51 ± 19^a^	0.91 ± 0.05^a^
GG03^a^^,^^b^	58 ± 1^a^	49 ± 24^a^	56 ± 17^a^	0.85 ± 0.04^a^
XG02^a^	34 ± 1^cd^	48 ± 22^a^	46 ± 15^a^	0.68 ± 0.05^b^
IC02^a^	29 ± 3^d^	42 ± 17^a^	53 ± 18^a^	0.52 ± 0.07^c^
XG04^b^	28 ± 4^d^	39 ± 14^a^	48 ± 18^a^	0.39 ± 0.01^cd^
IC055^b^	30 ± 4^d^	52 ± 25^a^	58 ± 17^a^	0.36 ± 0.02^d^

Values with a different letter within the same column are significantly different (P < 0.05).

a Group of ice cream mixes with similar mix viscosity.

b Group of ice cream mixes with similar serum phase viscosity.

Compared with the low-fat (1%) reference, increasing fat content and degree of fat destabilization did not appear to influence the overrun. This was consistent with our previous research, which also showed that fat content and degree of fat destabilization did not appear to have a significant effect on the overrun values. This had probably to do with a dominant effect of the freezing process on overrun in our model systems (Liu et al., 2022). For the samples with polysaccharides, the overrun of ice cream with XG and IC was similar to that of the low-fat reference (approximately 30%), while the overrun of LBG055 and GG03 was significantly higher, between 47 and 58% (P < 0.05). Flexible polysaccharides thus have a higher ability to incorporate air cells than rigid polysaccharides. A similar result was found in the study of Salahi & Mohebbi. (2021), which showed that GG and LBG led to higher overrun than XG in soy milk foams. This could be attributed to the lower shear-thinning behavior or more liquid-like viscoelastic behavior of samples with flexible polysaccharides. During ice cream preparation, shear stresses are experienced by the samples. In the case of ice cream with flexible polysaccharides, a higher viscosity is maintained at such higher stresses, which allows to retain the air in the sample. Under these conditions, the lower viscosity of samples with rigid polysaccharides as a result of the larger shear thinning behavior might lead to an easier escape of the air. In addition, the more liquid-like viscoelastic behavior of the flexible polysaccharides might provide sufficient molecular flexibility at the air-serum interfaces, which can result in more expansion of the air cells. Furthermore, we would like to highlight that the increase in mix viscosity resulting from adding polysaccharides did not always contribute to an increase in overrun, as samples with rigid polysaccharides showed overrun similar to that of the low-fat reference without polysaccharides. Instead, the structure of the polysaccharide appeared to play a significant role in determining the development of overrun.

In the present study, no significant differences were found in air cell size and ice crystal size among the studied samples (P > 0.05). In other studies, the size distribution of air pockets was influenced by the viscosity of the ice cream mix (Chang and Hartel, 2002; Sofjan and Hartel, 2004), which was not the case in our research. Small differences were observed, but they were not statistically significant. These results could be explained by our process conditions. It has been reported that the size of air cells and ice crystals can be strongly affected by the freezing conditions, including shear force, shear speed and drawing time etc. (Chang et al., 2002; Eisner et al., 2005; Muse et al., 2004; Scholten, 2014). As our samples were prepared with the same freezing process, the similar air cell and ice crystal size suggests that the freezing conditions might have played a more dominant role in determining air cell size and ice crystal size than small differences in viscosity. However, a significant difference was found in the ratio between the viscosity of ice cream mix and that of the molten ice cream, indicating that for some of the samples network formation occurred during freezing. An extremely low ratio (<0.01) was observed in high-fat molten ice cream. In addition, based on the fat particle size distribution in ice cream mix and molten high-fat ice cream (supplementary material of Fig. 1S), the D_4,3_ of the fat particles increased during ice cream preparation from 6.0 μm (ice cream mix) to 47.2 μm (molten ice cream). This value was larger than the critical fat aggregate size (approximately 45 μm) needed to provide a stable 3D fat network, which was obtained from our previous work (Liu et al., 2022). In addition, the fat aggregate percentage increased from 7% to 90%, which was also high enough to indicate fat network formation (Liu et al., 2022). These data indicate that a fat network was thus present in this sample. This network formation has been reported in previous studies (Granger et al., 2005; Liu et al., 2022). In comparison, limited network formation was obtained in the sample with 1% fat, as the viscosity ratio was 0.87. Such high values were also found for samples containing flexible polysaccharides, i.e., 0.55% LBG and 0.3% GG. However, a lower viscosity ratio between mix and molten ice cream was observed for samples with 0.2% xanthan gum and 0.2% iota carrageen, which can be attributed to a certain degree of gel network formation. In addition, more solid like properties were also obtained in samples with 0.4% xanthan and 0.55% iota carrageenan, which provided an additional indication that these samples most likely formed a network.

**Fig. 2:**
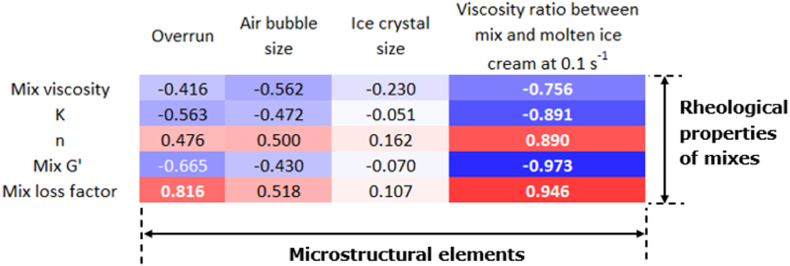
Heatmap of correlation matrix (Pearson coefficients) between rheological properties of ice cream mixes and microstructural elements of frozen ice cream (without high-fat sample). Blue indicates negative correlation and red indicates positive correlation. (For interpretation of the references to color in this figure legend, the reader is referred to the Web version of this article.)

To clarify which properties of the mix were related to the structure of ice cream, correlations between rheological properties of ice cream mixes and different microstructural characteristics of the final ice creams were determined. As shown in Fig. 2, overrun was highly correlated with the loss factor of the mix (0.816). The liquid-like behavior of the ice cream mix is thus the most important factor for incorporation of air cells. A lower correlation was found between overrun and mix viscosity (-0.416), indicating that the structural characteristics of the polysaccharides were more important in affecting overrun than mix viscosity. Both the air cell size and ice crystal size did not appear to be significantly (<-0.562) correlated with the rheological properties of ice cream mixes. As mentioned before, the freezing conditions, including scraper blade speed and drawing time, could play a more important role in the development of air cells and ice crystals. The viscosity ratio was negatively correlated with K (-0.891) and G' (-0.973), but positively correlated with n (0.890) and loss factor (0.946). The formation of a gel network is reflected by the rheological properties of ice cream mixes: an ice cream mix with a higher shear-thinning behavior and a more solid-like viscoelastic behavior had a higher ability to form a gel network in the serum phase during the freezing process.

In conclusion, ice cream mixes with flexible polysaccharides showed a higher ability to incorporate air cells due to their low shear sensitivity and molecular flexibility. Rigid polysaccharides provided a more solid-like behavior and tended to form a gel network in the serum phase, which was linked to less air incorporation.

#### 3.2.2 Rheological tribological, and textural properties

Viscoelastic behavior upon increasing temperature (G' at zone I (G'ZI) and slope of zone II (SZII)), tribological parameters (mean friction coefficient of the boundary regime (FCB) and slope of the mixed regime (SMR)), and textural properties (hardness and scooping energy) of the ice cream samples were determined, as they are important features relating to both the ice cream structure and sensory perception.

As shown in Table 6, no significant differences could be found in G'ZI and hardness between the references with low and high fat content. The reason why fat content had limited effect on these parameters was most likely the dominant effect of ice and air cells on hardness. As both the low-fat and high-fat references had the same ice fraction and similar overrun and ice crystal size, they thus had similar G'ZI and hardness. These results are consistent with the studies of Roland et al. (1999) and Rolon et al. (2017), in which it was also shown that the fat content did not significantly affect hardness. The addition of rigid polysaccharides (XG and IC) did not significantly affect G'ZI and hardness, but LBG055 and GG03 showed significantly lower hardness compared to the other samples, likely due to their higher overrun. An inverse relationship between hardness and overrun has been observed by many researchers (Biasutti et al., 2013; Muse et al., 2004; Sofjan et al., 2004). However, the overrun did not appear to affect scoopability, as we did not observe a higher scoopability, i.e., lower values of scooping energy, in LBG055 and GG03. However, XG04 and IC055 showed much higher values of scooping energy, and were thus more difficult to scoop. This could be due to the higher degree of gel network formation by XG and IC, which made it more difficult for a spoon to cut through the dense structure.

**Table 6:** Rheological (G' at zone I and slope of zone II), tribological (mean friction coefficient of boundary regime and slope of the mixed regime) and textural properties (hardness and scooping energy) of ice creams with different polysaccharides. The notation of the samples includes the name of the polysaccharide and the concentration used. For example, LBG055 refers to a sample with 0.55% LBG.

Ice cream	G' at zone I (10^6^ Pa, G'ZI)	Slope of zone II (SZII)	Mean friction coefficient of boundary regime (FCB)	Slope of the mixed regime (SMR)	Hardness (MPa)	Scooping energy (N · mm)
1% fat	5.4 ± 1.5^a^	-1.23 ± 0.01^d^	0.31 ± 0.01^ab^	-0.27 ± 0.01^d^	11.2 ± 1.8^a^	437 ± 24^c^
10% fat	5.8 ± 1.7^a^	-0.87 ± 0.03^a^	0.25 ± 0.01^cd^	-0.21 ± 0.01^c^	9.3 ± 0.4^a^	565 ± 98^bc^
LBG055^a^^,^^b^	4.8 ± 1.4^a^	-0.83 ± 0.01^a^	0.24 ± 0.01^d^	-0.16 ± 0.01^a^	3.2 ± 0.2^b^	564 ± 123^bc^
GG03^a^^,^^b^	4.7 ± 1.2^a^	-1.05 ± 0.01^b^	0.28 ± 0.01^bc^	-0.19 ± 0.01^ab^	2.8 ± 1.0^b^	605 ± 90^bc^
XG02^a^	5.7 ± 1.7^a^	-1.25 ± 0.02^d^	0.27 ± 0.01^cd^	-0.20 ± 0.01^bc^	6.9 ± 1.1^ab^	504 ± 63^c^
IC02^a^	5.9 ± 1.6^a^	-1.14 ± 0.01^c^	0.32 ± 0.01^a^	-0.18 ± 0.01^ab^	10.9 ± 2.3^a^	538 ± 68^bc^
XG04^b^	5.4 ± 1.5^a^	-0.89 ± 0.02^a^	0.26 ± 0.01^cd^	-0.20 ± 0.01^bc^	7.6 ± 0.7^ab^	915 ± 68^ab^
IC055^b^	5.6 ± 1.5^a^	-0.85 ± 0.02^a^	0.25 ± 0.01^cd^	-0.17 ± 0.01^ab^	9.2 ± 1.0^a^	1156 ± 195^a^

Values with a different letter within the same column are significantly different (P < 0.05).

a Group of ice cream mixes with similar mix viscosity.

b Group of ice cream mixes with similar serum phase viscosity.

The reference with high fat content (10% fat) and the samples with higher serum phase viscosity (LBG055, GG03, XG04 and IC055, denoted with b) showed significantly lower values of SZII, indicating that they melted more slowly. The lower melting in the high-fat (10%) reference could be attributed to the formation of a fat network formed by fat aggregates, which could block the continuous phase between air cells and create a more tortuous path for the serum phase to escape (Liu et al., 2022). In addition, the highly viscous serum phase created by the addition of polysaccharides also appeared to contribute to a slower melting rate. The high serum phase viscosity probably led to slow drainage of the serum becoming more and more diluted upon melting. However, the formation of a more solid-like network in the samples XG and IC did not have an additional contribution to melting, as their values of SZII were similar to those of LBG055. Therefore, the viscosity of the serum phase itself seemed to be more dominant.

We also observed differences in lubrication properties among samples. As expected, the 10% fat sample had a significantly lower friction coefficient in the boundary regime (FCB) and a lower slope of the mixed regime (SMR) compared with the 1% fat sample. As known, fat is a good lubricant, the addition of polysaccharides to the low fat (1%) sample decreased the friction coefficient, especially in the mixed regime. Also a significantly lower slope of the mixed regime was found after adding polysaccharides. In the case of the polysaccharides, the flexible LBG provided the lowest FCB and SMR. However, the lubricating capacity of the flexible polysaccharides (LBG and GG) was not significantly different from that of the rigid polysaccharides (XG and IC). This was unexpected, as it has been reported that stiff and charged polysaccharides provide better lubrication than flexible polysaccharides (Garrec and Norton, 2012). This could be attributed to the presence of various ingredients in the ice cream. Ice cream is a complex system, and the various ingredients and the interactions among them can affect the lubrication properties of the whole system, and overshadow the lubrication effect of the polysaccharides themselves.

**Fig. 3:**
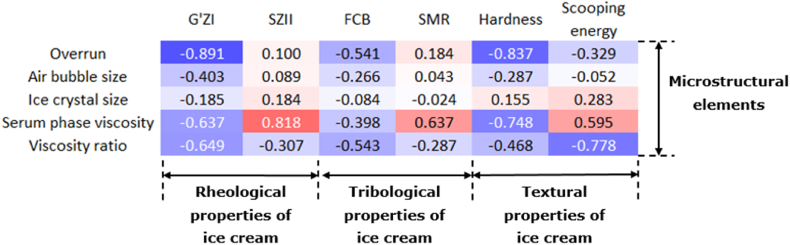
Heatmap of correlation matrix (Pearson coefficients) between microstructural elements and rheological, tribological and textural properties of the studied samples (without high-fat sample). Blue indicates negative correlation and red indicates positive correlation. (For interpretation of the references to color in this figure legend, the reader is referred to the Web version of this article.)

Correlation coefficients were determined to evaluate how microstructural elements affected the rheological and physical properties of ice cream with different polysaccharides (Fig. 3). Overrun was negatively correlated with G'ZI (-0.891) and hardness (-0.837): samples with a higher overrun had a more open structure, and were thus softer. Serum phase viscosity was negatively correlated to hardness (-0.748): in the mix viscosity series, samples prepared with flexible polysaccharides had a higher serum phase viscosity and a higher overrun. However, the correlation between these two parameters was not significant (0.478, data not shown). Scoopability (scooping energy) appeared to be negatively correlated with the viscosity ratio (-0.778): the formation of a gel network resulted in a more dense network, and therefore more energy was needed during scooping. All rheological parameters thus seemed more related to the structure of the polysaccharides than to their direct contribution to viscosity.

The melting properties were correlated to the serum phase viscosity, but was less affected by the type of polysaccharide. SZII showed a strong correlation with serum phase viscosity (0.818), but a relatively low correlation with the viscosity ratio, i.e., network formation (-0.307). No microstructural elements showed strong correlations with FCB and SMR. So, next to the fact that the type of polysaccharide was not linked to lubrication properties, also other microstructural elements of ice cream did not directly affect the lubrication behavior.

### 3.3 Sensory evaluation

#### 3.3.1 Sensory properties of ice cream

**Fig. 4:**
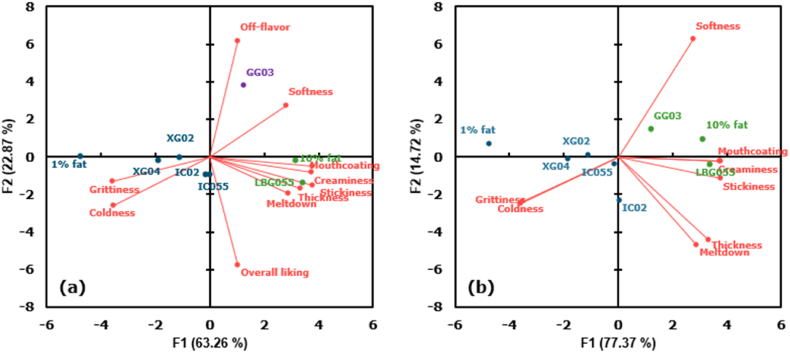
Principal Components Analysis of the studied ice cream samples with (a) all sensory attributes and (b) all sensory properties without off-flavor and overall liking, where the red points with lines refer to the different sensory properties and the dots with the other colors refer to the studied samples. Different colors of points indicate different groups. (For interpretation of the references to color in this figure legend, the reader is referred to the Web version of this article.)

The results of the sensory evaluation are shown in Table 7. Significant differences in all sensory attributes could be found among studied samples, as all P values were lower than 0.05. To identify common features of the samples, we also present a biplot obtained with Principal Components Analysis (PCA) (Fig. 4a). The first two principal components explained 86.13% of the variation. The first factor (F1, 63.26%) was mainly related to textural properties (creaminess, softness, coldness, grittiness, thickness, stickiness, mouth coating and meltdown), while the second factor (F2, 22.87%) was mainly linked to off-flavor and overall liking. The eight experimental samples were grouped into three groups, which are shown in different colors (Fig. 4a). The first one included the 10% fat reference and LBG055, and they were related to mouth coating, creaminess, thickness, stickiness and meltdown; the second group consisted of GG03 only, which had the strongest off-flavor; the third group included the 1% fat reference, XG02, XG04, IC02, and IC055, and they were most linked to grittiness and coldness. The fact that F2 was mainly related to flavor and overall liking only shows that off-flavor played a dominant role in determining the overall appreciation of the samples. The polysaccharide guar gum was mostly responsible for this off flavor. To better understand the effect of polysaccharides on sensory properties apart from the off-flavor, we removed off-flavor and overall liking from the analysis, and the obtained biplot is shown in Fig. 4b. We found that GG03 could then be categorized into the same group of LBG055 and 10% fat, indicating that LBG and GG had a similar effect on the texture of ice cream. Both flexible polysaccharides could provide textural properties similar to those of the high-fat sample, although the scores of the ice cream with LBG were closer to those of the full fat sample (Table 7). These samples were related to higher scores for mouth coating, creaminess, stickiness and thickness. On the other hand, the low-fat sample and the samples with rigid polysaccharides were located in the opposite direction, and showed the highest scores for grittiness and coldness. It has been reported before by Ares et al. (2010) that grittiness and coldness could be considered drivers of disliking, while creaminess is strongly related to liking. Flexible polysaccharides thus have a higher ability to improve the sensory properties of low-fat ice cream than rigid polysaccharides.

**Table 7:** Scores for the sensory properties of the studied samples. The notation of the samples includes the name of the polysaccharide and the concentration used. For example, LBG055 refers to a sample with 0.55% LBG.

Attributes	1% fat	10% fat	LBG055	GG03	XG02	IC02	XG04	IC055	F value	P Value
Creaminess	3.3 ± 1.9^d^	4.7 ± 1.9^ab^	5.2 ± 1.8^a^	4.3 ± 2.0^bc^	4.2 ± 2.2^bc^	4.1 ± 2.2^bc^	3.8 ± 2.0^cd^	4.1 ± 1.9^bc^	7.65	<0.01***
Softness	3.1 ± 1.8^cd^	4.8 ± 2.2^a^	4.1 ± 2.2^bc^	4.5 ± 1.9^ab^	3.5 ± 2.1^bc^	2.7 ± 1.9^d^	3.4 ± 2.0^bc^	3.6 ± 1.9^bc^	10.98	<0.01***
Coldness	6.6 ± 1.7^a^	5.3 ± 1.9^d^	5.5 ± 1.8^cd^	5.2 ± 1.5^d^	5.9 ± 1.9^bc^	5.9 ± 1.6^bc^	6.3 ± 1.5^ab^	6.0 ± 1.9^bc^	10.30	<0.01***
Grittiness	5.1 ± 2.3^a^	3.0 ± 2.3^c^	3.6 ± 2.3^bc^	3.5 ± 2.3^bc^	4.3 ± 2.6^ab^	4.3 ± 2.3^ab^	4.1 ± 2.5^ab^	4.0 ± 2.5^ab^	9.04	<0.01***
Thickness	3.3 ± 1.9^c^	4.2 ± 1.9^b^	4.6 ± 1.9^a^	4.0 ± 1.9^bc^	3.8 ± 2.0^c^	4.5 ± 2.3^ab^	3.7 ± 2.0^c^	4.0 ± 1.9^bc^	5.84	<0.01***
Stickiness	2.1 ± 1.6^d^	3.4 ± 2.2^ab^	3.6 ± 2.2^a^	2.8 ± 2.0^bc^	2.6 ± 2.1^cd^	2.9 ± 2.3^bc^	2.4 ± 2.0^cd^	2.9 ± 1.8^bc^	8.50	<0.01***
Mouth coating	3.3 ± 1.9^c^	4.5 ± 2.0^ab^	4.9 ± 2.0^a^	4.2 ± 2.1^ab^	3.7 ± 2.0^bc^	4.0 ± 2.1^bc^	3.6 ± 2.0^bc^	4.0 ± 1.9^bc^	7.60	<0.01***
Meltdown	3.9 ± 2.2^b^	4.7 ± 2.1^a^	4.6 ± 1.8^a^	4.4 ± 1.9^ab^	4.4 ± 2.1^ab^	4.8 ± 2.3^a^	4.5 ± 2.2^ab^	4.6 ± 2.0^a^	2.27	0.02*
Off-flavor	1.3 ± 1.8^b^	1.7 ± 1.9^b^	1.4 ± 1.8^b^	4.0 ± 3.0^a^	1.6 ± 2.0^b^	1.6 ± 2.1^b^	1.4 ± 1.9^b^	1.3 ± 1.7^b^	24.43	<0.01***
Overall liking	4.6 ± 1.8^ab^	5.2 ± 2.0^a^	5.3 ± 2.0^a^	3.5 ± 2.1^c^	4.7 ± 1.9^ab^	4.5 ± 1.9^ab^	4.6 ± 1.8^ab^	5.0 ± 1.8^a^	10.20	<0.01***

Values with a different letter within the same row are significantly different (P < 0.05).

**Fig. 5:**
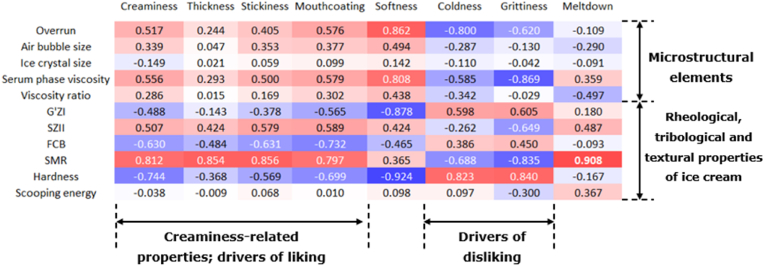
Heatmap of correlation matrix (Pearson coefficients) between physicochemical characteristics and sensory properties of the studied ice cream samples (without high-fat sample and the attributes off-flavor and overall liking). Blue indicates negative correlation and red indicates positive correlation. (For interpretation of the references to color in this figure legend, the reader is referred to the Web version of this article.)

To get a better understanding of how the sensory attributes were related to the properties of the ice cream, correlation coefficients between physicochemical characteristics and sensory properties are shown in Fig. 5. Off-flavor and overall liking were omitted from the analysis.

Some strong correlations were found between ice cream characteristics and sensory properties. As expected, overrun was strongly correlated with softness (0.862). This also explained the high negative correlation between G'ZI and softness (-0.878). In addition, overrun was negatively linked to coldness (-0.800). As air is a bad heat conductor, higher overrun leads to slower melting, which has been discussed before by Sofjan et al. (2004). Therefore, we also expected a high negative correlation between overrun and meltdown. However, a low correlation (-0.109) was found. This result is consistent with the findings of Wu et al. (2019) and Freire et al. (2020), who also found that overrun can only affect the meltdown under certain conditions. For example, Wu et al. (2019) found that overrun only had an effect on meltdown when no stabilizers were added. In our case, the low correlation between overrun and meltdown could be attributed to the addition of polysaccharides. Furthermore, the fast melting of our ice cream during consumption could be another reason for the difficulty to distinguish differences among samples. These results could indicate that coldness is not only related to the meltdown behavior, but also to other characteristics of the ice cream, such as the connectivity among ice crystals or structural changes of the serum phase. As overrun was related to the structure of the polysaccharides, we can conclude that polysaccharide structure also influences sensory perception.

Serum phase viscosity was highly correlated with softness (0.808) and grittiness (-0.869). Therefore, these attributes did not seem to be influenced much by the type of polysaccharides, but just by the viscosity of the serum phase. The positive correlation between serum phase viscosity and softness could be attributed to the fact that a higher serum phase viscosity seemed to be linked to a higher overrun. In addition, the fact that grittiness was inversely related to serum viscosity could be explained by the fact that a more viscous serum phase can mask the detection of ice crystals during oral processing (Clarke, 2015). Although grittiness is often related to larger ice crystal sizes (Clarke, 2003), we did not see a correlation between these two parameters. This is however logical, as the ice crystal size in our samples was not significantly different, and therefore other aspects became more important.

Although the lubrication properties were not directly linked to structural changes in the ice cream, they were correlated with several sensory attributes. The slope of the mixed regime (SMR) was positively correlated with creaminess (0.812), thickness (0.854), stickiness (0.856), and mouth coating (0.797). These are sensory attributes for which lubrication properties indeed play a role. However, it is worth noting that the friction coefficient in the boundary regime (FCB) did not appear to be closely correlated with any of these sensory attributes, indicating that SMR seems to be more relevant. In previous studies, sensory attributes have always been linked to friction coefficients at a specific velocity, i.e. FCB, and correlations have indeed been observed (Chojnicka-Paszun et al., 2014; Fan et al., 2021; Upadhyay and Chen, 2019). However, our results showed that this is not the case for ice cream, which may be related to its complex structure. Ji et al. (2022) also found that sensory perception cannot always be explained based on one single friction coefficient extracted from a Stribeck curve, and that dynamic changes in the friction coefficient during different stages of oral processing should also be taken into account. Such dynamic changes are represented in the parameter SMR. As the structure of ice cream during oral processing is affected by the melting of the ice crystals and a dilution of the serum phase and subsequent decrease in viscosity, it is not surprising that the parameter SMR correlates better with sensory perception than FCB. Although we expected a high correlation between thickness and serum phase viscosity, and melting rate and sensory meltdown, these correlations were rather low (0.293 and 0.487, respectively). These results could indicate that thickness and meltdown are affected by multiple factors, which need further investigation.

In conclusion, flexible polysaccharides showed a higher ability to improve the sensory properties of low-fat ice cream than rigid polysaccharides. This seems mostly related to an increased overrun, which was directly correlated to increased softness and reduced coldness. Grittiness perception did not seem to be affected by the type of polysaccharides, but was mainly affected by serum phase viscosity. Attributes as creaminess, thickness and mouthcoating were more related to lubrication aspects (SMR), but could not be linked to the structure of the polysaccharides.

## 4 Conclusion

In this study, we investigated the effect of polysaccharide structure on the rheological, tribological, textural and sensory properties of ice cream. Flexible polysaccharides (LBG and GG) were less shear-thinning and showed a more liquid-like viscoelastic behavior compared with rigid polysaccharides (XG and IC), which was reflected in differences in the properties of the ice cream mix. Mixes with flexible polysaccharides showed a higher ability to incorporate air cells, while rigid polysaccharides provided a more solid-like behavior and tended to form a gel network in the serum phase, related to less air incorporation. The higher overrun of samples containing flexible polysaccharides led to lower hardness, while ice creams with rigid polysaccharides were harder to scoop. Overrun and scoopability were thus affected by the type of polysaccharide. However, melting rate was not related to the type of polysaccharide, but to serum phase viscosity. Also, no link between polysaccharide type and lubrication performance was found, nor did other structural ice cream characteristics directly link to lubrication parameters. Based on sensory evaluation, two main groups were found: ice creams with flexible polysaccharides were closer to the full-fat reference ice cream, while those with rigid polysaccharides were closer to the low-fat reference ice cream. Ice creams with flexible polysaccharides were associated with higher softness and creaminess-related properties, while rigid polysaccharides were related to higher coldness. Some attributes did not seem to be directly related to the type of polysaccharide, but to other characteristics. For example, grittiness was more related to serum viscosity, while creaminess, thickness and mouthcoating were related to changes in the friction coefficient. These results indicate that flexible polysaccharides can be considered more suitable fat replacers for improving the sensory properties of low-fat ice cream than rigid polysaccharides.

## CRediT authorship contribution statement

**Xiangyu Liu:** Investigation, Data curation, Methodology, Visualization, Writing – original draft. **Guido Sala:** Methodology, Conceptualization, Supervision, Writing – review & editing, Funding acquisition. **Elke Scholten:** Methodology, Conceptualization, Supervision, Writing – review & editing, Funding acquisition.

## Declaration of competing interest

The authors declare that no competing interest exists.

## Data Availability

Data will be made available on request.

## References

[bib1] Akbari M., Eskandari M.H., Davoudi Z. (2019). Application and functions of fat replacers in low-fat ice cream: a review. Trends Food Sci. Technol..

[bib2] Alvarez V.B. (2009).

[bib3] Amador J., Hartel R., Rankin S. (2017). The effects of fat structures and ice cream mix viscosity on physical and sensory properties of ice cream. J. Food Sci..

[bib4] Ares G., Giménez A., Barreiro C., Gámbaro A. (2010). Use of an open-ended question to identify drivers of liking of milk desserts. Comparison with preference mapping techniques. Food Qual. Prefer..

[bib5] BahramParvar M., Tehrani M.M., Razavi S.M.A. (2013). Effects of a novel stabilizer blend and presence of κ-carrageenan on some properties of vanilla ice cream during storage. Food Biosci..

[bib6] Bai L., Liu F., Xu X., Huan S., Gu J., McClements D.J. (2017). Impact of polysaccharide molecular characteristics on viscosity enhancement and depletion flocculation. J. Food Eng..

[bib7] Biasutti M., Venir E., Marino M., Maifreni M., Innocente N. (2013). Effects of high pressure homogenisation of ice cream mix on the physical and structural properties of ice cream. Int. Dairy J..

[bib8] Chang Y., Hartel R.W. (2002). Development of air cells in a batch ice cream freezer. J. Food Eng..

[bib9] Chojnicka-Paszun A., Doussinault S., De Jongh H.H.J. (2014). Sensorial analysis of polysaccharide–gelled protein particle dispersions in relation to lubrication and viscosity properties. Food Res. Int..

[bib10] Clarke C. (2003). The physics of ice cream. Phys. Educ..

[bib11] Clarke C. (2015).

[bib12] Dogan M., Aslan D., Gurmeric V. (2018). The rheological behaviors and morphological characteristics of different food hydrocolloids ground to sub-micro particles: in terms of temperature and particle size. J. Food Meas. Char..

[bib13] Eisner M.D., Wildmoser H., Windhab E.J. (2005). Air cell microstructuring in a high viscous ice cream matrix. Colloids Surf. A Physicochem. Eng. Asp..

[bib14] Fan N., Shewan H.M., Smyth H.E., Yakubov G.E., Stokes J.R. (2021). Dynamic Tribology Protocol (DTP): response of salivary pellicle to dairy protein interactions validated against sensory perception. Food Hydrocolloids.

[bib15] Ferdiansyah R., Abdassah M., Zainuddin A., Rachmaniar R., Chaerunisaa A.Y. (2023). Effects of alkaline solvent type and pH on solid physical properties of carrageenan from Eucheuma cottonii. Gels.

[bib16] Freire D.O., Wu B., Hartel R.W. (2020). Effects of structural attributes on the rheological properties of ice cream and melted ice cream. J. Food Sci..

[bib17] Fuhrmann P.L., Sala G., Scholten E., Stieger M. (2020). Influence of clustering of protein-stabilised oil droplets with proanthocyanidins on mechanical, tribological and sensory properties of o/w emulsions and emulsion-filled gels. Food Hydrocolloids.

[bib18] Garrec D.A., Norton I.T. (2012). The influence of hydrocolloid hydrodynamics on lubrication. Food Hydrocolloids.

[bib19] Goff H.D., Hartel R.W. (2013).

[bib20] Granger C., Leger A., Barey P., Langendorff V., Cansell M. (2005). Influence of formulation on the structural networks in ice cream. Int. Dairy J..

[bib21] Hadinugroho W., Martodihardjo S., Fudholi A., Riyanto S. (2021). Preparation of citric acid-locust bean gum (CA-LBG) for the disintegrating agent of tablet dosage forms. J. Pharmaceut. Innovat..

[bib22] Javidi F., Razavi S.M.A., Behrouzian F., Alghooneh A. (2016). The influence of basil seed gum, guar gum and their blend on the rheological, physical and sensory properties of low fat ice cream. Food Hydrocolloids.

[bib23] Ji L., den Otter D., Cornacchia L., Sala G., Scholten E. (2023). Role of polysaccharides in tribological and sensory properties of model dairy beverages. Food Hydrocolloids.

[bib24] Ji L., Orthmann A., Cornacchia L., Peng J., Sala G., Scholten E. (2022). Effect of different molecular characteristics on the lubrication behavior of polysaccharide solutions. Carbohydr. Polym..

[bib25] Kurt A., Cengiz A., Kahyaoglu T. (2016). The effect of gum tragacanth on the rheological properties of salep based ice cream mix. Carbohydr. Polym..

[bib26] Liu X., Sala G., Scholten E. (2022). Effect of fat aggregate size and percentage on the melting properties of ice cream. Food Res. Int..

[bib27] Mahdian E., Karazhian R. (2013). Effects of fat replacers and stabilizers on rheological, physicochemical and sensory properties of reduced-fat ice cream. J. Agric. Sci. Technol..

[bib28] Meyners M., Jaeger S.R., Ares G. (2016). On the analysis of rate-all-that-apply (RATA) data. Food Qual. Prefer..

[bib29] Milani E., Koocheki A. (2011). The effects of date syrup and guar gum on physical, rheological and sensory properties of low fat frozen yoghurt dessert. Int. J. Dairy Technol..

[bib30] Muse M.R., Hartel R.W. (2004). Ice cream structural elements that affect melting rate and hardness. J. Dairy Sci..

[bib31] Petri D.F.S. (2015). Xanthan gum: a versatile biopolymer for biomedical and technological applications. J. Appl. Polym. Sci..

[bib32] Picout D.R., Ross-Murphy S.B., Jumel K., Harding S.E. (2002). Pressure cell assisted solution characterization of polysaccharides. 2. Locust bean gum and tara gum. Biomacromolecules.

[bib33] Pintor A., Escalona-Buendía H.B., Totosaus A. (2017). Effect of inulin on melting and textural properties of low-fat and sugar-reduced ice cream: optimization via a response surface methodology. Int. Food Res. J..

[bib34] Roland A.M., Phillips L.G., Boor K.J. (1999). Effects of fat content on the sensory properties, melting, color, and hardness of ice cream. J. Dairy Sci..

[bib35] Rolon M.L., Bakke A.J., Coupland J.N., Hayes J.E., Roberts R.F. (2017). Effect of fat content on the physical properties and consumer acceptability of vanilla ice cream. J. Dairy Sci..

[bib36] Rosti M.E., Takagi S. (2021). Shear-thinning and shear-thickening emulsions in shear flows. Phys. Fluids.

[bib37] Salahi M.R., Mohebbi M. (2021). Development of soy milk in the form of wet foam in the presences of whey protein concentrate and polysaccharides at different whipping temperatures: study of physical, rheological and microstructural properties. LWT.

[bib38] Scholten E. (2014). Particulate Products.

[bib39] Sofjan R.P., Hartel R.W. (2004). Effects of overrun on structural and physical characteristics of ice cream. Int. Dairy J..

[bib40] Soukoulis C., Chandrinos I., Tzia C. (2008). Study of the functionality of selected hydrocolloids and their blends with κ-carrageenan on storage quality of vanilla ice cream. LWT--Food Sci. Technol..

[bib41] Stokes J.R., Macakova L., Chojnicka-Paszun A., de Kruif C.G., de Jongh H.H.J. (2011). Lubrication, adsorption, and rheology of aqueous polysaccharide solutions. Langmuir.

[bib42] Taira Y., McNamee C.E. (2014). Polysaccharide films at an air/liquid and a liquid/silicon interface: effect of the polysaccharide and liquid type on their physical properties. Soft Matter.

[bib43] Thompson K.R., Chambers D.H., Chambers Iv E. (2009). Sensory characteristics of ice cream produced in the USA and Italy. J. Sensory Stud..

[bib44] Thrimawithana T.R., Young S., Dunstan D.E., Alany R.G. (2010). Texture and rheological characterization of kappa and iota carrageenan in the presence of counter ions. Carbohydr. Polym..

[bib45] Upadhyay R., Chen J. (2019). Smoothness as a tactile percept: correlating ‘oral’tribology with sensory measurements. Food Hydrocolloids.

[bib46] Velásquez-Cock J., Serpa A., Vélez L., Gañán P., Hoyos C.G., Castro C., Duizer L., Goff H.D., Zuluaga R. (2019). Influence of cellulose nanofibrils on the structural elements of ice cream. Food Hydrocolloids.

[bib47] Wildmoser H., Scheiwiller J., Windhab E.J. (2004). Impact of disperse microstructure on rheology and quality aspects of ice cream. LWT--Food Sci. Technol..

[bib48] Wu B., Freire D.O., Hartel R.W. (2019). The effect of overrun, fat destabilization, and ice cream mix viscosity on entire meltdown behavior. J. Food Sci..

