# Supplementary material for: Role of polysaccharide structure in the rheological, physical and sensory properties of low-fat ice cream
Source: Curr Res Food Sci. 2023 Jun 11;7:100531. doi: 10.1016/j.crfs.2023.100531 (PMC10333429; doi:10.1016/j.crfs.2023.100531)
Supplement: Multimedia component 1 [file mmc1.docx]

Fig. 1S. Fat particle size distribution of ice cream mix (black line) and molten high-fat ice cream (red line). The D_4,3_ of ice cream mix and molten ice cream was 6.0 and 47.2 µm, and the fat aggregate percentage was 7% and 90%, respectively.
